# Utilization of Integrated Child Development Services (ICDS) and its linkages with undernutrition in India

**DOI:** 10.1111/mcn.13644

**Published:** 2024-04-08

**Authors:** Shri K. Singh, Alka Chauhan, Harold Alderman, Rasmi Avula, Laxmi K. Dwivedi, Rati Kapoor, Trupti Meher, Purnima Menon, Phuong H. Nguyen, Sarang Pedgaonker, Parul Puri, Suman Chakrabarti

**Affiliations:** ^1^ International Institute for Population Sciences Mumbai India; ^2^ The International Food Policy Research Institute Washington District of Columbia USA

**Keywords:** child development, child health services, child malnutrition, nutrition interventions, nutrition policy, nutritional status, underweight

## Abstract

The Integrated Child Development Services (ICDS) programme has been the central focus of the POSHAN Abhiyaan to combat maternal and child malnutrition under the national nutrition mission in India. This paper examined the linkages between utilization of ICDS and underweight among children aged 6–59 months. The study utilized data from two recent rounds of the National Family Health Survey (NFHS‐4 [2015–2016] and NFHS‐5 [2019–2021]). Descriptive analyses were used to assess the change in utilization of ICDS and the prevalence of underweight at the national and state levels. Multivariable logistic regressions were performed to examine factors associated with the utilization of ICDS and underweight. Linkages between utilization of ICDS and underweight were examined using the difference‐in‐differences (DID) approach. Utilization of ICDS increased from 58% in 2015–2016 to 71% in 2019–2021. The prevalence of underweight decreased from 37% to 32% in the same period. Changes in ICDS utilization and underweight prevalence varied considerably across states, socioeconomic and demographic characteristics. Results from decomposition of DID models suggest that improvements in ICDS explained 9%–12% of the observed reduction in underweight children between 2016 and 2021, suggesting that ICDS made a modest but meaningful contribution in addressing undernutrition among children aged 6–59 months in this period.

## Key messages

- Utilization of ICDS services among children aged 6–59 months increased from 58% (2015–2016) to 71% (2019–2021) nationally. The programme's expansion was prominent in urban areas, showcasing progress across states.
- The prevalence of underweight among children in India declined by 5 percentage points from 2016 to 2021, but almost one‐third of children are still underweight.
- Increase in coverage and service delivery strengthening of ICDS explained 9%–12% of the observed reduction in underweight between 2016 and 2021.
- The findings suggest that optimizing quality of interventions under ICDS can have a modest but meaningful contribution in reducing childhood underweight in India.

## Graphical abstract

This study examines linkages between Integrated Child Development Services (ICDS) utilization and underweight in 6–59 months old children in India, using nationally representative data. ICDS utilization increased by 13 pp and underweight prevalence decreased by 5 pp during 2016–2021. Improved ICDS coverage accounts for 9%–12% of observed underweight reduction, signifying a modest yet meaningful role in addressing child undernutrition.

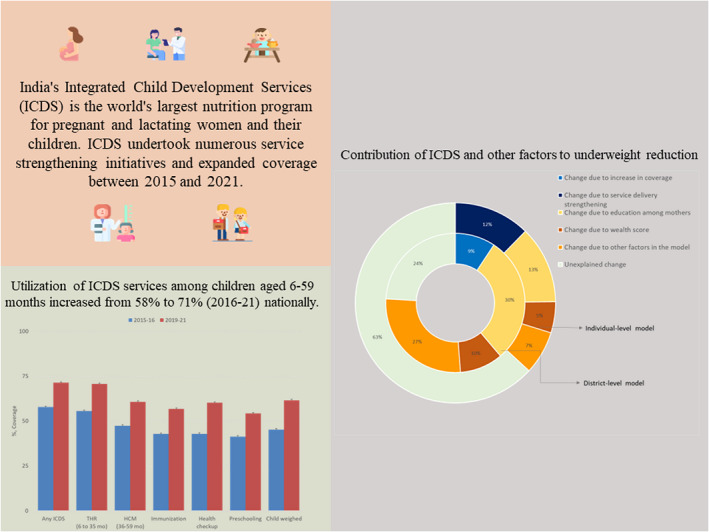

## 1 INTRODUCTION

Child undernutrition, characterized by stunting, wasting and underweight, remains high in many low‐ and middle‐income countries. Stunting and underweight contribute to 20% of all child mortality among children younger than 5 years of age (Black et al., 2013). In an effort to achieve the Sustainable Development Goals (SDGs), there is enhanced attention to all forms of malnutrition and this presents an opportune time to address undernutrition.

In India, nearly a third of children are underweight, reducing slowly from 43% to 32% in the last 15 years (IIPS & ICF, 2007, 2017, 2022). Multiple factors, including those at child, maternal and household levels, are associated with underweight across several countries. These factors include fetal growth restriction (Black et al., 2013), low dietary diversity, partial vaccination, maternal underweight (Corsi et al., 2016; Li et al, 2020), short maternal stature, low maternal education (Li et al, 2020), early marriage and low socioeconomic status (Corsi et al., 2016; Li et al, 2020).

India's Integrated Child Development Services (ICDS) is the largest early childhood programme in the world, providing services to pregnant and lactating women and children below 6 years of age (Ministry of Women and Child Development, 2013b). The programme offers supplementary nutrition, preschool education, nutrition and health education, and growth monitoring, and supports the health department in the implementation of immunization, basic health checkups and referral services (Ministry of Women and Child Development, 2013b).

Several studies have evaluated the effectiveness of the ICDS in enhancing maternal and child health in India ever, as it was first implemented. Even though the programme has been running smoothly for several decades, there are divergent views on the degree of its success (Jain, 2015; Kandpal, 2011). The evidence is sparse and mixed on the impact of ICDS on the nutritional status of children. There is limited evidence on its impact on overall child nutrition status (Gupta et al., 2005), but there is significant treatment effect of ICDS among the most malnourished children (Kandpal, 2011). Studies conducted at the state level did not find significant effect of ICDS enrolment and receipt of supplemental food under ICDS on the prevalence of underweight among children (Dutta & Ghosh, 2017; Thakur et al., 2011). However, daily supplementary feeding was associated with better nutritional status of young children in rural areas (Jain & Agnihotri, 2020); nutrition and health education from the ICDS had no significant nutritional benefits for children (Dixit et al., 2018).

Between 2016 and 2021, there were substantial changes in the quality of ICDS services provided. The initiation of the POSHAN Abhiyaan (PA) in 2018 bolstered the ICDS, universalizing it and integrating it with other nutrition‐focussed programmes for women and children (NITI Aayog, 2019). The PA also encompassed the National Early Childhood Care and Education policy introduced in 2013, which aimed at the holistic development of children aged 0–6 years. As part of the PA, investments were also made in the umbrella ICDS programme, which integrated Early Childhood Care and Education with the National Creche Scheme and Child Protection Scheme. Under the PA, the government implemented several other policy actions to strengthen the existing programmes (Ministry of Women and Child Development, 2013b). This included revising the cost norms for supplementary nutrition, with increased allocations sanctioned in September 2017 for beneficiaries of Anganwadi services under ICDS. Furthermore, incentives for frontline workers were enhanced. To strengthen growth monitoring and enhance decision‐making, the government embraced technological advancements through the implementation of the ICDS‐CAS (Common Application Software) under the PA. This software enables effective monitoring, timely interventions and data‐driven decision‐making. Moreover, various nongovernmental organizations supported the delivery of ICDS in districts across India with a high burden of malnutrition, by providing financial assistance, human resources and infrastructural support (World Bank, 2023).

Given the multiple strategies implemented to strengthen ICDS between 2016 and 2021, it is opportune to examine whether improvements in undernutrition occurred concurrently. Although it is challenging to disentangle the effect of these qualitative interventions, their collective implementation has likely contributed to improving the efficacy of ICDS overall. As a result, this study seeks to (1) document the extent of utilization of ICDS services across the continuum of services between 2016 and 2021, (2) examine changes in the prevalence of underweight among under‐five children and (3) explore the association of coverage expansion and implicit qualitative improvements within the ICDS on the prevalence of underweight among children in India.

## 2 METHODS

### 2.1 Data source

We used child‐ and household‐level data from the two recent rounds of the Indian National Family Health Survey (NFHS‐4, 2015–2016 and NFHS‐5, 2019–2021) (IIPS & ICF, 2017, 2022). NFHS is designed to provide national and subnational estimates of various critical indicators to monitor the SDGs on population, health, nutrition and gender equality, among others. These cross‐sectional surveys follow a systematic, multistage stratified sampling design and are representative at both the state and district levels in India. This study utilized data of the youngest child aged 6–59 months (*n* = 214,972 children from NFHS‐4 and 198,335 from NFHS‐5). Youngest child refers to most recent births of the respondent women. All statistical analyses were performed with STATA 17.0 (StataCorp) and MS Excel. All estimates were reported by applying appropriate sampling weights provided by the NFHS.

### 2.2 Ethical considerations

The Institutional Review Board at IIPS granted NFHS ethical approval after they adhered to all necessary regulations. No additional ethical approval was needed for utilising the existing NFHS data.

### 2.3 Outcome variables

We examined two key variables in this study. The first variable measures the utilization of any ICDS service during childhood. Further, we selected childhood underweight as the primary outcome, aligning with one of the key target indicators outlined in the ICDS framework (Ministry of Women and Child Development, 2013a). Children's weight was measured using a standard technique by well‐trained enumerators. A weight‐for‐age *z*‐score (WAZ) was derived by comparing each child's anthropometric measurements to the World Health Organization (WHO) age‐ and gender‐appropriate child growth standards (de Onis & WHO Multicentre Growth Reference Study Group, 2006). WAZ below −2 SD from the median was considered underweight per WHO recommendation (de Onis & WHO Multicentre Growth Reference Study Group, 2006).

### 2.4 Explanatory variables

Explanatory variables were considered at household, maternal and child levels. Household‐level factors include place of residence, enrolment into health insurance, household size, religion, social groups (caste/tribes) and wealth. A household wealth index was constructed using principal component analysis of multiple variables including house and land ownership, housing structure and ownership of assets and livestock. The wealth score was divided into quintiles across survey rounds to obtain an ordinal measure of household wealth relative to other households that was consistent across time (Filmer & Pritchett, 2001). Mother‐level factors include age (years), educational level (years) and height (cm). Child‐level factors include age (year), sex and birth order. We also included district fixed effects to control for time invariant district‐level characteristics.

### 2.5 Analytical strategy

#### 2.5.1 Correlates of ICDS and underweight prevalence

We first used descriptive analyses to document the changes in utilization of any ICDS‐specific services and underweight prevalence at the national and state levels and stratified by socioeconomic and demographic characteristics. We then employed regression analyses to examine the association between explanatory variables and utilization of any ICDS services or underweight prevalence using the following equation:

(1) $$\text{Logistic}\;\left(P\left(Y=1|x_{1},x_{2},\text{…},x_{k}\right)\right)=(\beta_{0}+\sum \beta_{i}x_{i}),$$

where *Y* is the outcome of interest and *x*_i_ represents the set of explanatory variables.

These regression analyses identified the set of variables that are associated with both ICDS service utilization and underweight, controlled for potential confounders.

#### 2.5.2 Difference‐in‐differences (DID) approach

Linkages between the utilization of any ICDS and child underweight were assessed using DID models (Villa, 2016). DID methods involve the use of panel data or repeated cross‐sections (Fredriksson & Oliveira, 2019). We pooled repeated cross‐sections from NFHS‐4 and ‐5, because the NFHS follows the same states and districts over time, thus enabling longitudinal analysis at the district levels. DID requires at least two time periods and two treatment groups (Wing et al., 2018). In our analyses, the two time periods are 2015–2016 and 2019–2021, and the treatment group includes those receiving any ICDS services, whereas the control group includes those who do not receive any services.

The association between ICDS utilization and underweight was estimated at two levels: (1) at the individual level, to distinguish the effect of change due to service delivery strengthening and (2) at the district level, to estimate the contribution of change due to increase in coverage. At the individual level, we estimated Equation (2) (Kishore & Chakrabarti, 2015) for child *i*, from household *h*, district *d* and year of survey *t*:

(2) $$Y_{\text{ihdt}}=\beta_{0}+\beta_{1}{\mathrm{ICDS}}_{ihdt}+\beta_{2}T_{t}+\beta_{3}{\mathrm{ICDS}}_{ihdt}\;*\;T_{t}+\beta_{4}C_{ihdt}\;*\;T_{t}+D_{d}+\varepsilon_{ihdt}\text{…},$$

where ICDS is the treatment dummy that takes value 0 for the control group and 1 for the treated group, and *T* takes the value 0 for NFHS4 and 1 for NFHS5. $\beta_{0}$ is the baseline prevalence of underweight, $\beta_{1}$ is the difference between the control and treated group at baseline, $\beta_{2}$ captures the time trend in the control group and $\beta_{3}$ indicates the difference in the changes between ICDS nonusers and users over the period of 2016–2021. $\beta_{4}$ indicates coefficients of each of the explanatory variable, $C_{\mathrm{ihdt}}$, discussed above. Lastly, $D_{d}$ represents district fixed effects and $\epsilon_{\mathrm{ihdt}}$ is the residual term. SEs are clustered at the district level to account for intradistrict correlations.

At the district level, we used aggregate figures for child underweight and utilization of any services under ICDS for districts. We estimated Equation (3) for district *d* and year of survey *t* (Chakrabarti et al., 2021):

(3) $$\begin{array}{ccl} Y_{dt} & = & \gamma_{0}+\gamma_{1}{\mathrm{ICDS}}_{dt}+\gamma_{2}T_{t}+\gamma_{3}{\mathrm{ICDS}}_{dt}\;*\;T_{t}\\ & & +\;\gamma_{4}C_{dt}\;*\;T_{t}+D_{d}+\epsilon_{dt}\text{…}, \end{array}$$

where ICDS is the treatment dummy, aggregated at district level, is a continuous variable that captures proportion of children utilizing any service under ICDS. $\gamma_{3}$, the DID estimate, represents the additional change in underweight per unit coverage of ICDS over the period of 2016–2021.

At both the individual and district levels, our DID results are based on endogenous treatment choices. In other words, ICDS beneficiaries are likely to be systematically different from nonbeneficiaries because they self‐select into using the programme rendering our estimates susceptible to bias from unobserved factors.

#### 2.5.3 Preintervention parallel trends

The underlying assumption for DID to be valid is that that preintervention parallel trends were present in the outcome before 2015. To test this assumption, we used data from NFHS‐3 (2005–2006) and NFHS‐4. We plot trends in underweight between 2015 and 2016 to gauge if prevalence of child underweight prevalence moved in tandem across the treatment and control groups. We estimated these results using equation (2) where $T_{t}$ represents linear trend and takes value 1 for 2005–2006 and 10 for 2015–2016 (preintervention period). The validation of preintervention parallel trends lends confidence to our DID model estimates.

#### 2.5.4 Postintervention accelerating trends

To check the robustness of the DID estimator, we test for accelerating trends among children receiving services from ICDS (Fredriksson & Oliveira, 2019). Interrupted time series models were used to test whether the pace of reductions in underweight prevalence increased in the period 2016–2021 compared to the period 2006–2015 for a subsample of ICDS beneficiaries (Bernal et al., 2016).

#### 2.5.5 Regression‐based decomposition

We performed regression‐based decomposition with the DID coefficient $\beta_{3}$ from Equation (3).

We estimated the effect of coverage changes in ICDS between 2016 and 2021 using Equation (4) (Chakrabarti et al., 2021).

(4) $$\frac{\beta_{3}(\mathrm{ICDS}\;{\mathrm{coverage}}_{2021})}{\mathrm{Child}\;\mathrm{underweight}\;{\mathrm{prevalence}}_{2015}-\mathrm{Child}\;\mathrm{underweight}\;{\mathrm{prevalence}}_{2021}}\ldots$$

We also employed an alternative methodology to estimate the population‐level effect of service delivery strengthening in ICDS between 2016 and 2021 based on $\gamma_{3}\;$as indicated in Equation (5).

(5) $$\frac{\gamma_{3}{(\mathrm{change}\;\mathrm{in}\;\mathrm{ICDS}\;\mathrm{coverage}\;\mathrm{among}\;\mathrm{beneficiaries}}_{2016-2021})}{\mathrm{Child}\;\mathrm{underweight}\;{\mathrm{prevalence}}_{2016}-\mathrm{Child}\;\mathrm{underweight}\;{\mathrm{prevalence}}_{2021}}\ldots$$

The results in Equation (4) show the impact of increased coverage of ICDS. However, Equation (5) indicates the contribution of service delivery strengthening within the ICDS.

#### 2.5.6 Sensitivity analysis

We executed the primary individual‐level DID model from Equation (2) on two distinct subsamples: states surveyed during coronavirus disease 2019 (COVID‐19) and those surveyed before the pandemic. This examination aimed to discern potential variations in ICDS utilization associated with the influence of COVID‐19.

#### 2.5.7 Robustness checks

For robustness checks, we modified the primary individual‐level DID model from Equation (2) to include treatment groups reflecting frequencies of food supplementation. We added two treatments—frequent (at least weekly) food supplementation and less frequent (monthly or less) food supplementation as indicated in Equation (6).

(6) $$\begin{array}{ccl} Y_{ihdt} & = & \beta_{0}+\beta_{1}{\mathrm{Frequent}\;\mathrm{food}\;\mathrm{supp}}_{ihdt}\\ & & +\;\beta_{2}{\mathrm{Less}\;\mathrm{frequent}\;\mathrm{food}\;\mathrm{supp}}_{ihdt}\\ & & +\;\beta_{3}T_{t}+\beta_{4}{\mathrm{Frequent}\;\mathrm{food}\;\mathrm{supp}}_{ihdt}\;*\;T_{t}\\ & & +\;\beta_{5}\mathrm{Less}\;{\mathrm{frequent}\;\mathrm{food}\;\mathrm{supp}}_{ihdt}\;*\;T_{t}\\ & & +\;\beta_{6}C_{ihdt}\;*\;T_{t}+D_{d}+\varepsilon_{ihdt}\ldots . \end{array}$$

Additionally, we used the individual DID model (Equation [2]) to ascertain the impact of ICDS on stunting and wasting. This allowed us to assess the programme's influence on additional anthropometric indicators. Furthermore, we explored the coverage of food supplementation across various receipt frequencies over the two survey rounds. Finally, we ran our primary individual‐level DID model (Equation [2]) on subsamples of rural and urban residences to test for heterogeneity by place of residence.

## 3 RESULTS

**Figure 1:**
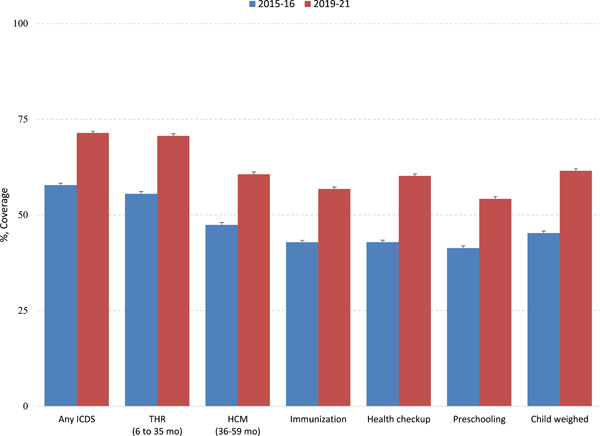
Utilization of various services under ICDS for Indian children aged 6–59 months for the period 2015–2016 to 2019–2021. ICDS, Integrated Child Development Services; HCM, hot cooked meals; THR, take home ration. All estimates are adjusted for sampling weights.

**Figure 2:**
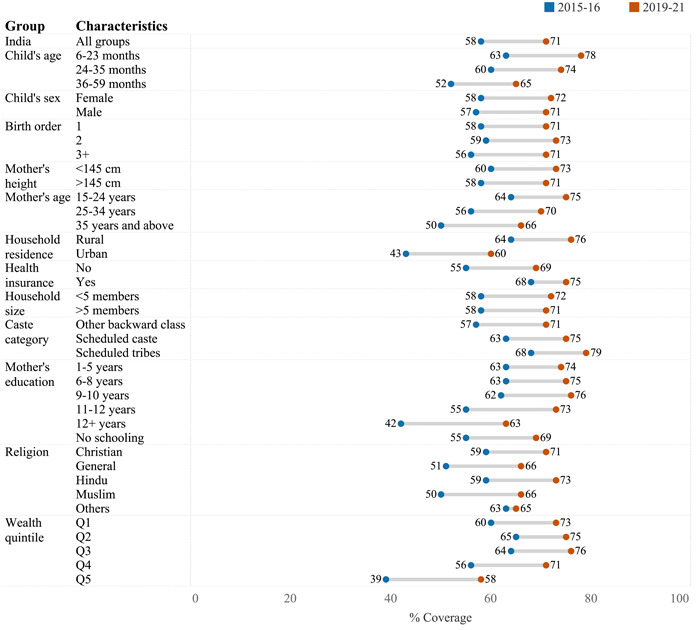
Utilization of any child‐specific services under ICDS for Indian children aged 6–59 months stratified by covariates for the period 2015–2016 to 2019–2021. ICDS, Integrated Child Development Services. Q1 refers to the bottom wealth quintile and Q5 refers to the top wealth quintile. All estimates are adjusted for sampling weights.

Figure 1 shows the coverage of various services under ICDS for children of the age group 6–59 months across the survey years 2015–2016 and 2019–2021. There is a notable increase in coverage across all services, with health checkups showing the most substantial growth—an increase of 17 percentage points (pps). Figure 2 depicts the utilization of any ICDS services by background characteristics across NFHS‐4 and NFHS‐5. Utilization has increased from 58% in 2015–2016 to 71% in 2019–2021 at the national level; this improvement was observed in almost every state, with Andhra Pradesh, Chhattisgarh, Karnataka, Madhya Pradesh and Odisha being the best performers (Supporting Information S1: Table S1). Among household‐level factors, although coverage was high in rural areas, the increase in coverage was higher in urban areas than that in rural areas. The change from 2015–2016 to 2019–2021 was much higher among children belonging to mothers aged 35 years and above, mothers with schooling 12 years and above, Muslim households and highest wealth quintile (Supporting Information S1: Table S2). Drivers of ICDS utilization did not change much between 2016 and 2021 (Table 1); the common explanatory variables include child's age, mother's schooling, religion, caste, residence and wealth index.

**Table 1:** Association between background characteristics and utilization of any ICDS or underweight prevalence among children aged 6–59 months, 2015–2021.

	Any service received from ICDS during childhood				Underweight prevalence			
	2015–16 (*N* = 205,918)		2019–21 (*N* = 186,650)		2015–16 (*N* = 205,918)		2019–21 (*N* = 186,650)	
Covariates	AOR	95% CI	AOR	95% CI	AOR	95% CI	AOR	95% CI
Child age								
6–23 months (ref)	1.00	[1.00,1.00]	1.00	[1.00,1.00]	1.00	[1.00,1.00]	1.00	[1.00,1.00]
24–35 months	0.86***	[0.84,0.89]	0.85***	[0.82,0.88]	1.25***	[1.22,1.28]	1.23***	[1.19,1.27]
36–59 months	0.61***	[0.58,0.64]	0.53***	[0.51,0.56]	1.27***	[1.23,1.30]	1.23***	[1.20,1.27]
Child's sex								
Female (ref)	1.00	[1.00,1.00]	1.00	[1.00,1.00]	1.00	[1.00,1.00]	1.00	[1.00,1.00]
Male	0.98	[0.96,1.00]	0.98	[0.96,1.00]	1.08***	[1.06,1.10]	1.11***	[1.08,1.13]
Birth order								
1 (ref)	1.00	[1.00,1.00]	1.00	[1.00,1.00]	1.00	[1.00,1.00]	1.00	[1.00,1.00]
2	1.03**	[1.01,1.06]	1.06***	[1.03,1.08]	1.13***	[1.10,1.16]	1.14***	[1.11,1.17]
3+	0.99	[0.96,1.03]	1.00	[0.96,1.05]	1.23***	[1.18,1.27]	1.25***	[1.21,1.30]
Mother's age								
15–24 years (ref)	1.00	[1.00,1.00]	1.00	[1.00,1.00]	1.00	[1.00,1.00]	1.00	[1.00,1.00]
25–34 years	0.95**	[0.92,0.98]	1.01	[0.96,1.05]	0.87***	[0.84,0.89]	0.90***	[0.88,0.93]
35 years and above	0.86***	[0.81,0.91]	0.96	[0.91,1.02]	0.77***	[0.73,0.81]	0.79***	[0.76,0.83]
Mother's height								
<145 cm (ref)	1.00	[1.00,1.00]	1.00	[1.00,1.00]	1.00	[1.00,1.00]	1.00	[1.00,1.00]
≥145 cm	0.97	[0.93,1.01]	0.99	[0.94,1.04]	0.54***	[0.52,0.55]	0.54***	[0.52,0.56]
Mother's schooling								
No schooling (ref)	1.00	[1.00,1.00]	1.00	[1.00,1.00]	1.00	[1.00,1.00]	1.00	[1.00,1.00]
1–5 years	1.34***	[1.27,1.41]	1.25***	[1.18,1.33]	0.88***	[0.85,0.91]	0.90***	[0.87,0.94]
6–8 years	1.41***	[1.33,1.50]	1.32***	[1.24,1.40]	0.81***	[0.78,0.84]	0.86***	[0.82,0.90]
9–10 years	1.42***	[1.32,1.53]	1.36***	[1.27,1.45]	0.68***	[0.65,0.72]	0.78***	[0.75,0.82]
11–12 years	1.19***	[1.10,1.30]	1.25***	[1.16,1.35]	0.64***	[0.60,0.67]	0.70***	[0.67,0.74]
12+ years	0.90*	[0.83,0.98]	0.99	[0.91,1.08]	0.53***	[0.50,0.57]	0.62***	[0.58,0.65]
Residence								
Rural (ref)	1.00	[1.00,1.00]	1.00	[1.00,1.00]	1.00	[1.00,1.00]	1.00	[1.00,1.00]
Urban	0.58***	[0.54,0.63]	0.58***	[0.54,0.62]	1.12***	[1.07,1.16]	1.08***	[1.03,1.13]
Health insurance								
No (ref)	1.00	[1.00,1.00]	1.00	[1.00,1.00]	1.00	[1.00,1.00]	1.00	[1.00,1.00]
Yes	1.68***	[1.55,1.82]	1.32***	[1.23,1.42]	0.98	[0.94,1.02]	0.98	[0.95,1.02]
Household size								
<5 members (ref)	1.00	[1.00,1.00]	1.00	[1.00,1.00]	1.00	[1.00,1.00]	1.00	[1.00,1.00]
**≥**5 members	0.98	[0.95,1.02]	1.01	[0.97,1.05]	1.04**	[1.02,1.07]	1.05**	[1.02,1.08]
Religion								
Others (ref)	1.00	[1.00,1.00]	1.00	[1.00,1.00]	1.00	[1.00,1.00]	1.00	[1.00,1.00]
Hindu	1.11	[0.91,1.36]	2.06***	[1.74,2.45]	1.36***	[1.18,1.57]	1.53***	[1.34,1.75]
Muslim	0.79*	[0.64,0.99]	1.52***	[1.27,1.82]	1.24**	[1.06,1.46]	1.63***	[1.41,1.88]
Christian	0.64**	[0.48,0.86]	1.04	[0.82,1.33]	0.62***	[0.51,0.76]	0.84*	[0.71,1.00]
Caste category								
General (ref)	1.00	[1.00,1.00]	1.00	[1.00,1.00]	1.00	[1.00,1.00]	1.00	[1.00,1.00]
Scheduled caste	1.38***	[1.29,1.48]	1.28***	[1.21,1.37]	1.32***	[1.26,1.39]	1.27***	[1.20,1.33]
Scheduled tribes	1.45***	[1.29,1.63]	1.37***	[1.23,1.54]	1.35***	[1.24,1.46]	1.26***	[1.17,1.36]
Other backward classes	1.19***	[1.11,1.27]	1.18***	[1.10,1.25]	1.28***	[1.21,1.34]	1.19***	[1.14,1.25]
Wealth quintile								
Poorest Q1 (ref)	1.00	[1.00,1.00]	1.00	[1.00,1.00]	1.00	[1.00,1.00]	1.00	[1.00,1.00]
Q2	1.12***	[1.05,1.19]	1.05	[0.99,1.11]	0.77***	[0.74,0.80]	0.79***	[0.76,0.82]
Q3	1.14**	[1.05,1.23]	1.12**	[1.04,1.22]	0.62***	[0.60,0.65]	0.69***	[0.66,0.73]
Q4	1.02	[0.92,1.12]	1.09	[1.00,1.19]	0.51***	[0.49,0.54]	0.57***	[0.54,0.61]
Richest Q5	0.77***	[0.68,0.87]	0.82***	[0.74,0.91]	0.44***	[0.41,0.47]	0.46***	[0.43,0.49]

*Note*: Estimates are calculated using the logistic regression model where outcome is underweight prevalence among children aged 6–59 months. All estimates are adjusted for sampling weights. Q1 refers to the bottom wealth quintile and Q5 refers to the top wealth quintile.

Abbreviations: AOR, adjusted odds ratio; CI, confidence interval; ICDS, Integrated Child Development Services.

* *p* < 0.05

** *p* < 0.01

*** *p* < 0.001.

**Figure 3:**
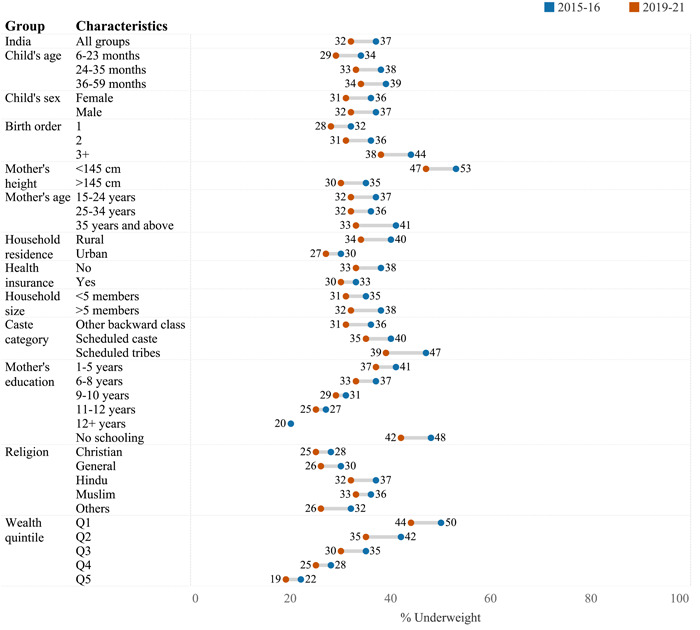
Prevalence of underweight among Indian children aged 6–59 months stratified by covariates for the period 2015–2016 to 2019–2021. ICDS, Integrated Child Development Services. Q1 refers to the bottom wealth quintile, and Q5 refers to the top wealth quintile. All estimates are adjusted for sampling weights.

**Figure 4:**
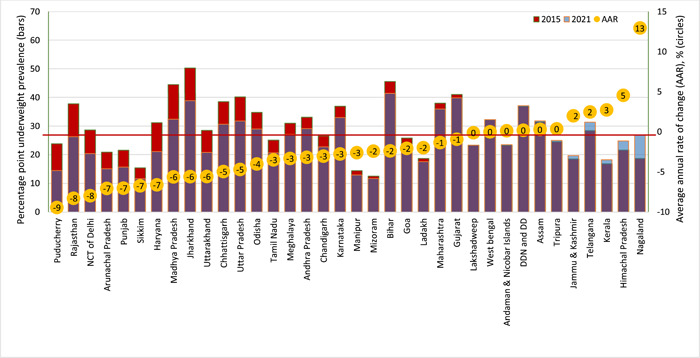
Underweight prevalence (absolute) and average annual rate of change (relative) across Indian states and union territories. The bars represent underweight prevalence (%) for 36 Indian states and union territories during NFHS‐4 (2015–2016) and NFHS‐5 (2019–2021) surveys. Each bubble corresponds to state‐specific average annual rate (AAR) of change in underweight prevalence over the entire period. The AAR was computed by comparing the underweight prevalence values (%) between NFHS‐4 and NFHS‐5, considering the number of years in the study period. A negative AAR indicates an average annual decrease, whereas a positive AAR indicates an average annual increase.

Figure 3 depicts the prevalence of underweight based on various characteristics across NFHS‐4 and NFHS‐5. The overall underweight prevalence has decreased from 37% in 2015–2016 to 32% in 2019–2021. A large variation was found in the annual average rate of change in underweight across states/UTs. States such as Nagaland, Himachal Pradesh, Kerala and Telangana documented a high rate of change (Figure 4). The prevalence of underweight was higher in rural areas, lowest wealth households, lower caste groups, higher birth orders and among children of uneducated mothers (Supporting Information S1: Table S3).

Factors associated with the prevalence of underweight among children were similar across two survey rounds. The odds of being underweight were higher among children of age groups 24–35 months and 36–59 months, higher birth order, belonging to Muslim households, greater family size and lower caste group compared to their counterparts. In contrast, the odds of being underweight were lower among children born to older mothers (aged 25 years and above), mothers with some educational attainment, Christian households and those belonging to affluent households (Table 1).

Table 2 provides the results of DID models from Equation (2). The DID coefficient in Model 1 showed 1 pp larger reduction in underweight prevalence among ICDS users between 2016 and 2021 compared to non‐ICDS users (*p* < 0.05). DID coefficients from Models 2 and 3 showed that the improvements were largely driven by nonfood ICDS services (−1.55 pp, *p* < 0.05). Regression coefficients from a subsample of unsplit districts were larger in magnitude but directionally similar. District‐level results from Equation (3) (Table 3) showed that underweight prevalence was 0.04 pp lower for every 1 pp increase in ICDS coverage. This association was larger (0.08 pp) and statistically significant in the unsplit district subsample (*p* < 0.05).

**Table 2:** Individual‐level analysis.

	Underweight prevalence, percentage points			
	All India sample		Unsplit districts subsample	
	*β*	95% CI	*β*	95% CI
Model 1: Any ICDS service				
Posttreatment period, %	−3.97***	[−4.76, −3.17]	−3.71***	[−4.55, −2.88]
Treatment group, %	1.73***	[1.19, 2.26]	1.77***	[1.22, 2.33]
Interaction (DID), %	−0.88**	[−1.66, −0.11]	−1.20**	[−2.03, −0.38]
Model 2: Nonfood ICDS services				
Posttreatment period, %	−3.65***	[−4.48, −2.82]	−3.45***	[−4.31, −2.58]
Treatment group, %	1.91***	[1.36, 2.45]	1.99***	[1.42, 2.56]
Interaction (DID), %	−1.44**	[−2.26, −0.62]	−1.71***	[−2.56, −0.87]
Model 3: Food‐based ICDS services				
Posttreatment period, %	−4.33***	[−5.08, −3.58]	−4.15***	[−4.94, −3.35]
Treatment group, %	1.41***	[0.88, 1.95]	1.48***	[0.92, 2.02]
Interaction (DID), %	−0.38	[−1.14, 0.37]	−0.64	[−1.43, 0.16]
Model specifications				
All covariates	Yes		Yes	
District fixed effects	Yes		Yes	
Clustered SEs at district level		Yes		Yes
*N* (number of children)	395,073		342,830	

*Note*: Regression‐based DIDs estimates for association between receiving services from ICDS and underweight prevalence among children aged 6–59 months, 2015–2021. Posttreatment period = dummy variable for 2019–2021; treatment group refers to any ICDS in Model 1, nonfood services from ICDS in Model 2, food‐based services from ICDS in Model 3; estimates are calculated using ordinary least squares model where outcome is underweight (see Equation [2]). Binary outcome was multiplied by 100 and can be interpreted as a percentage point difference. Covariates include child age, sex, birth order, maternal age, maternal height, maternal education, household residence, health insurance, household size, religion, caste and wealth. Coefficients not shown for brevity.

Abbreviations: CI, confidence interval; DID, difference‐in‐differences estimate; ICDS, Integrated Child Development Services.

*p < 0.05

** *p* < 0.01

*** *p* < 0.001.

**Table 3:** District‐level analysis.

	Underweight prevalence			
	OLS regression		GLS regression	
	*β*	95% CI	*β*	95% CI
Model 1: All Indian sample (640 districts), *N* = 1280				
Posttreatment period	−0.51	[−3.66, 2.64]	−0.77	[−3.77, 2.23]
District ICDS coverage, %	0.14***	[0.11, 0.18]	0.11***	[0.08, 0.15]
Interaction (DID), %	−0.04	[−0.09, 0.00]	−0.03	[−0.08, 0.01]
Model 2: Unsplit districts subsample (575 districts), *N* = 1150				
Posttreatment period	0.02	[−0.01, 0.05]	0.02	[−0.01, 0.05]
District ICDS coverage, %	0.15***	[0.12, 0.19]	0.13***	[0.09, 0.16]
Interaction (DID), %	−0.08**	[−0.12, −0.03]	−0.07**	[−0.11, −0.03]
Model specifications				
All covariates	Yes		Yes	
District fixed effects	No		Yes	
Clustered SE at district level	Yes		Yes	

*Note*: Regression‐based DID estimates for association between receiving any ICDS with underweight among children aged 6–59 months, 2015–2021. Posttreatment period = dummy variable for 2019–2021; district ICDS coverage was calculated by collapsing means and adjusting for sampling weights for each district using pooled NFHS‐4 and NFHS‐5 data. Estimates are calculated using ordinary least square model (see Equation [3]). Covariates include child age, sex, birth order, maternal age, maternal height, maternal education, household residence, health insurance, household size, religion, caste and wealth

Abbreviations: CI, confidence interval; DID, difference‐in‐differences estimate; ICDS, Integrated Child Development Services; OLS, ordinary least squares; GLS, generalized least squares.

*p < 0.05

** *p* < 0.01

*** *p* < 0.001.

The assumption of preintervention parallel trends was validated for our main model (Supporting Information S1: Table S4). In robustness checks, interrupted time series models showed that ICDS beneficiaries experienced a faster reduction, compared to the pre‐2015 period, suggesting accelerating prevalence reduction trends associated with service strengthening (Supporting Information S1: Table S5).

**Figure 5:**
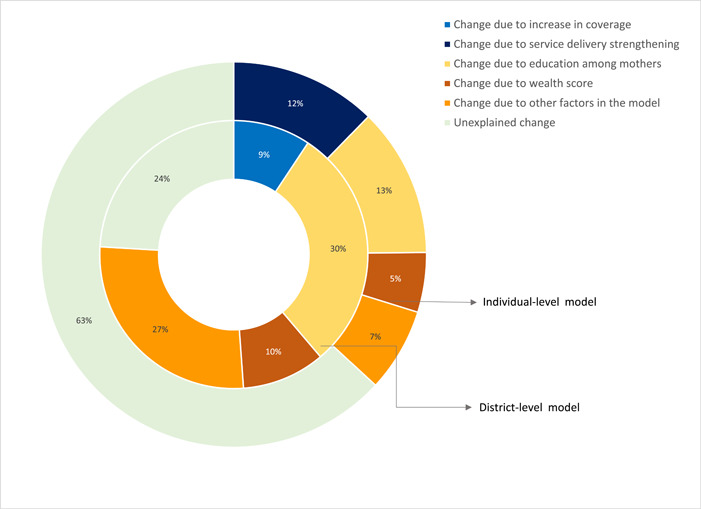
Decomposition of difference‐in‐differences estimate (DID) estimates explaining contribution of ICDS towards underweight reduction in India between 2015–2016 and 2019–2021. Outer ring presents individual model. Inner ring presents district‐level model. The 5 pp change in underweight prevalence among children aged 6–59 months between 2015–2016 and 2019–2021 (see Figure 2) is decomposed using coefficients from two regression models. (1) Change due to delivery strengthening is from individual‐level DID model (see Table 2) where the treatment is qualitative interventions to strengthen ICDS (any service) between 2016 and 2021, and outcome is underweight (see Equation [4]). (2) Change due to increase in coverage is from the district‐level model (see Table 3), where the treatment is coverage of any service under ICDS and outcome is underweight at the district level (see Equation [5]). ICDS, Integrated Child Development Services.

Figure 5 presents the result of the regression‐based decomposition of DID at the district and individual level. In both the results depicted in the figure, we also include the measured contribution of wealth and education based on the respective coefficients in Equations (2) and (3). As is readily apparent, trends in education contribute extensively to the reduction in underweight. ICDS service strengthening was associated with 12% of the observed change in the national prevalence of underweight (5 pp), whereas increases in ICDS coverage across districts accounted for 9% of the observed change. Although these percentages are not additive, overall improvements in ICDS contributed to changes in underweight in a similar magnitude as changes in wealth.

Additional analyses on stunting and wasting revealed that improvements in ICDS were associated with larger reductions in wasting (−0.91 pp, *p* < 0.05) but not associated with stunting (Supporting Information S1: Tables S6 and S7).

We conducted a sensitivity analysis (Supporting Information S1: Table S8) to explore the influence of the pandemic on ICDS utilization. Post‐COVID, food supplementation became significant in reducing underweight, whereas it was not significant in the pre‐COVID model. Conversely, nonfood services under ICDS were significant in the pre‐COVID model but lost significance in the latter model. Over the time there has been little change in distribution of receiving food supplementation under ICDS (Supporting Information S1: Table S9). However, to provide nuanced insights into food supplementation, we conducted robustness checks (Supporting Information S1: Table S10) for the DID model, utilizing food supplementation frequencies as treatments. The analysis reveals a significant and heightened impact of frequent supplementation, particularly evident in the during COVID‐19 sample.

Additional analysis stratifying results by place of residence revealed that associations were larger in rural areas (−1.02 pp, *p* < 0.05) than those in urban areas (0.05 pp, *p* > 0.05) (Supporting Information S1: Table S11).

## 4 DISCUSSION

Our study shows increased utilization of ICDS services by 13 pp at the national level between 2016 and 2021, although the results vary by socioeconomic and demographic characteristics. At the same time, prevalence of underweight declined by 5 pp among children, although it was variable by state. The reduction in underweight was 1 pp higher among those using ICDS compared to those who did not. This was likely due to strengthening of ICDS services and improvements in coverage across districts, as demonstrated by 12% and 9% contributions, respectively, to change in prevalence of underweight at the national level.

### 4.1 Utilization of ICDS services

ICDS service utilization improved across wealth, residential and demographic groups, associated with mother's age, education, place of residence and wealth quintile. Rural areas exhibited significantly higher utilization, consistent with the study by Kumar et al. (2022). In addition, mothers’ higher education correlated positively with ICDS utilization, emphasizing the role of education in raising awareness (Ghosh & Das, 2013; Kumar et al., 2022) and therefore underscores the need to promote women's education.

Furthermore, children from the highest wealth quintiles showed a lesser likelihood of service utilization. This could stem from perceptions of ICDS having low service quality among the economically sound households. Another possible explanation could be that affluent class may avoid ICDS services due to perceived stigma, as these services are commonly linked to poverty, creating a psychological barrier since they may be seen as intended for the less privileged.

### 4.2 Prevalence of underweight among children aged 6–59 months and its key drivers

Between 2016 and 2021, underweight prevalence decreased by 5pps; yet, approximately one‐third of children remain underweight. The annual average rate of change in underweight was lower from 2016 to 2021 compared to 2006–2016 (Varghese et al., 2022). State‐level variations suggest diverse underlying determinants (Avula et al., 2022).

Aligned with global research (Hossain et al., 2023; Kumar et al., 2019; Moshi et al., 2022; Tosheno et al., 2017), our study indicates that underweight likelihood rises with age, possibly due to insufficient supplementary feeding post 6 months or increased exposure to environmental hazards (Rao, 2011). The lower prevalence among girls updates past evidence on son preferences in India (Hvistendahl, 2011); recent data refute gender‐based nutritional discrepancies (Alderman et al., 2021).

Likelihood of being underweight increase with younger, less educated mothers and higher birth orders, aligning with prior research (Corsi et al., 2016; Kumar et al., 2019; Moshi et al., 2022; Sen et al., 2011; Wemakor et al., 2018). Educated mothers contribute to healthier diets and better access to health services (Murarkar et al., 2020).

Our findings reveal reduced undernutrition risk among under‐five children in rural areas, contrasting earlier urban‐centric studies (Bharati et al., 2008). Rural children benefit from access to fresh, organic and seasonal foods, promoting nutrient‐dense intake (Pradhan & Shete, 2022). Additionally, children in higher wealth quintiles are less likely to be underweight due to superior access to diverse diets, health care and improved living conditions (Chowdhury et al., 2018).

### 4.3 Linkages between ICDS and undernutrition

Previous nationally representative studies conducted before the ICDS reforms yielded mixed findings regarding the impact of the ICDS on underweight prevalence. Jain (2015) used matching methods and reported improved WAZs among boys aged 0–2 years receiving daily supplementary feeding, whereas Dixit et al. (2018) found no significant association between ICDS services during pregnancy and underweight. With the postreform universality of ICDS access across India, matching methods no longer provide suitable counterfactuals for ICDS beneficiaries due to the role of unobservable self‐selection. Thus, observational studies have shown an insignificant and sometimes negative association between ICDS utilization and undernutrition among under‐five children in the postreform period (Dixit et al., 2018; Pradhan & Shete, 2022). However, our DID analysis, accounting for preintervention parallel trends, reveals a small but significant correlation between ICDS services and underweight prevalence in 2019–2021 compared to 2015–2016.

Although the NFHS data lack explicit information on service quality, it is noteworthy that the Poshan Abhiyaan indicates improvements in quality over the two survey periods. Consequently, the indicators within the NFHS data suggest a more pronounced emphasis on quality in 2019–2021. These trends underscore the potential influence of qualitative changes and advancements ushered in by the PA initiative on the observed shifts in service quality measures between the two periods. Service delivery strengthening and coverage expansion contributed to 9%–12% of the overall reduction in national underweight rates during this period. Concurrently, improvements in maternal education accounted for a substantial 13%–30%, while changes in household wealth contributed additional 5%–10% to overall reduction in underweight. Our estimates are within the expected range of reductions predicted by global models on scaling‐up nutrition interventions (Black et al., 2013).

The COVID‐specific DID model shows an expanded role for ICDS as a safety net during pandemics and other potential shocks. However, it's crucial to acknowledge possible systematic differences between pre‐ and post‐COVID states that may have driven the differences in the results. Unfortunately, limitations in NFHS data, such as lacking district‐level pandemic severity information, hindered a more rigorous analysis of effect of COVID‐19 on ICDS.

### 4.4 Strengths and limitations

Our study addresses a crucial knowledge gap by examining the relationship between ICDS and underweight among children in India. By analysing comprehensive national data sets, we observed changes in ICDS service utilization and underweight rates. Causal inference, supported by DID models and confounder control, strengthens our findings. In addition, using decomposition models, we converted the estimated DID results into measurable changes in underweight prevalence. However, to precisely measure the impact of ICDS on underweight and other malnutrition outcomes, a designated impact evaluation with panel data collection is necessary with a clear control group, a condition which is difficult given the extensive coverage. The DID models validate the existence of preintervention parallel trends; however, the treatment and control groups still have different intercepts, suggesting that they differ on characteristics. The limited decline in underweight, coupled with high utilization among impoverished groups, suggests that our findings from observational data should be interpreted with caution regarding causality. Moreover, given the concurrent nature of coverage expansion and service delivery strengthening, our DID models were unable to isolate their individual contributions.

## 5 CONCLUSION

The findings of this study shed light on the modest but noteworthy role of the ICDS programme in curbing underweight prevalence among Indian children. To enhance this positive trajectory, sustained financial investment and a reinforced implementation framework are imperative. Equally crucial is a multisectoral approach that tackles the root causes of undernutrition. It is likely that integrating these components can amplify the efficacy of ICDS in combating childhood underweight.

## AUTHOR CONTRIBUTIONS

Shri K. Singh conceived the idea, led statistical analysis and reviewed the manuscript. Alka Chauhan conducted statistical analysis, drafted the initial manuscript and reviewed the manuscript. Harold Alderman, Rasmi Avula and Phuong H. Nguyen critically reviewed the manuscript and contributed to writing the manuscript. Laxmi K. Dwivedi, Sarang Pedgaonker and Trupti Meher reviewed manuscript and contributed to writing the manuscript. Rati Kapoor and Parul Puri. conducted statistical analysis and reviewed results for accuracy. Purnima Menon assisted conceptualization and intellectual insights. Suman Chakrabarti conducted statistical analysis, wrote significant sections and reviewed the manuscript. All authors read and approved the final manuscript as submitted.

## CONFLICT OF INTEREST STATEMENT

The authors declare no conflict of interest.

## Supporting information

Supporting Information

## Data Availability

The data that support the findings of this study are openly available in India: Standard DHS, 2019‐21 at https://dhsprogram.com/methodology/survey/survey-display-541.cfm. The data are available online on the website of the Demographic Health Survey.
