# Supplementary material for: Utilization of Integrated Child Development Services (ICDS) and its linkages with undernutrition in India
Source: Matern Child Nutr. 2024 Apr 8;20(3):e13644. doi: 10.1111/mcn.13644 (PMC11168363; doi:10.1111/mcn.13644)
Supplement: Supplementary file 1 — Supporting Information [file MCN-20-e13644-s001.docx]

**Supplementary information for-**

**Utilization of Integrated Child Development Services (ICDS) and its linkages with undernutrition in India**

**Table S1: Utilization of any service under the ICDS by Indian states and Union Territories during 2015-21.**

|  | **NFHS-4 (2015-16)** | | **NFHS-5 (2019-21)** | |
| --- | --- | --- | --- | --- |
|  | **Utilization, *%*** | | **Utilization, *%*** | |
| **India** | **57.80** | **[57.33, 58.26]** | **71.40** | **[70.95, 71.86]** |
| Andaman & Nicobar Islands | 54.19 | [45.35, 62.78] | 58.17 | [52.32, 63.81] |
| Andhra Pradesh | 76.23 | [73.97, 78.36] | 83.01 | [80.97, 84.87] |
| Arunachal Pradesh | 22.88 | [20.66, 25.27] | 38.26 | [35.67, 40.91] |
| Assam | 59.40 | [57.38, 61.38] | 71.25 | [69.53, 72.91] |
| Bihar | 52.06 | [50.78, 53.33] | 57.30 | [55.87, 58.72] |
| Chandigarh | 59.65 | [47.75, 70.51] | 59.27 | [47.84, 69.77] |
| Chhattisgarh | 82.65 | [81.23, 83.99] | 83.57 | [81.95, 85.07] |
| Dadra Nagar Haveli & Daman & Diu | 45.54 | [38.63, 52.63] | 70.84 | [65.21, 75.89] |
| Goa | 63.49 | [56.08, 70.32] | 76.92 | [71.68, 81.43] |
| Gujarat | 64.95 | [62.65, 67.18] | 72.57 | [70.64, 74.42] |
| Haryana | 47.45 | [44.90, 50.02] | 72.92 | [70.82, 74.91] |
| Himachal Pradesh | 78.08 | [75.42, 80.52] | 80.85 | [78.45, 83.05] |
| Jammu & Kashmir | 35.82 | [33.64, 38.05] | 52.21 | [49.83, 54.59] |
| Jharkhand | 60.66 | [59.15, 62.14] | 70.08 | [68.52, 71.58] |
| Karnataka | 64.73 | [62.45, 66.95] | 84.59 | [82.81, 86.22] |
| Kerala | 56.10 | [53.04, 59.11] | 64.51 | [61.82, 67.12] |
| Ladakh | 49.31 | [41.77, 56.89] | 64.78 | [57.63, 71.33] |
| Lakshadweep | 67.07 | [61.14, 72.51] | 58.24 | [48.43, 67.44] |
| Madhya Pradesh | 68.39 | [67.28, 69.48] | 84.44 | [83.47, 85.37] |
| Maharashtra | 52.19 | [49.90, 54.47] | 61.60 | [59.03, 64.10] |
| Manipur | 31.43 | [29.10, 33.85] | 44.84 | [41.44, 48.29] |
| Meghalaya | 58.83 | [55.69, 61.91] | 63.13 | [58.78, 67.27] |
| Mizoram | 70.10 | [66.03, 73.88] | 71.90 | [68.72, 74.88] |
| Nagaland | 37.15 | [34.27, 40.12] | 60.49 | [57.08, 63.80] |
| NCT of Delhi | 16.60 | [12.52, 21.68] | 60.07 | [56.34, 63.68] |
| Odisha | 82.90 | [81.61, 84.13] | 92.85 | [91.94, 93.67] |
| Puducherry | 63.17 | [54.66, 70.93] | 69.30 | [60.32, 77.03] |
| Punjab | 63.46 | [60.62, 66.21] | 56.08 | [53.50, 58.63] |
| Rajasthan | 41.87 | [40.41, 43.35] | 63.22 | [61.64, 64.78] |
| Sikkim | 53.84 | [49.33, 58.30] | 72.22 | [66.13, 77.59] |
| Tamil Nadu | 64.87 | [62.73, 66.95] | 80.79 | [79.05, 82.42] |
| Telangana | 63.54 | [59.75, 67.18] | 77.72 | [75.27, 79.98] |
| Tripura | 65.93 | [61.12, 70.43] | 76.14 | [73.12, 78.92] |
| Uttar Pradesh | 42.19 | [41.22, 43.16] | 70.52 | [69.51, 71.51] |
| Uttarakhand | 62.56 | [60.37, 64.70] | 77.26 | [75.22, 79.17] |
| West Bengal | 78.04 | [76.01, 79.94] | 82.61 | [80.51, 84.53] |

**Table S2. Utilization of any child specific services under ICDS for Indian children aged 6-59 months stratified by covariates for the period 2015-16 to 2019-21**

|  | | | | |
| --- | --- | --- | --- | --- |
| **Covariates** | **NFHS-4 (2015-16)** | | **NFHS-5 (2019-21)** | |
|  | **Utilization (%)** | **95% CI** | **Utilization (%)** | **95% CI** |
| **Child age, *months*** |  |  |  |  |
| 6-23 months | 63.42 | [62.79, 64.04] | 77.85 | [77.25, 78.43] |
| 24-35 months | 59.63 | [58.87, 60.39] | 74.02 | [73.29, 74.74] |
| 36-59 months | 52.47 | [51.89, 53.05] | 65.35 | [64.80, 65.91] |
| **Child’s sex, *%*** |  |  |  |  |
| Male | 58.20 | [57.63, 58.76] | 71.72 | [71.17, 72.26] |
| Female | 57.43 | [56.87, 57.98] | 71.11 | [70.57, 71.65] |
| **Birth order, *%*** |  |  |  |  |
| 1 | 57.85 | [57.23, 58.46] | 70.88 | [70.26, 71.48] |
| 2 | 59.22 | [58.57, 59.86] | 72.54 | [71.94, 73.13] |
| 3+ | 56.13 | [55.44, 56.82] | 70.74 | [70.04, 71.43] |
| **Mother's age, *y*** |  |  |  |  |
| 15-24 years | 63.79 | [63.08, 64.50] | 75.27 | [74.60, 75.94] |
| 25-34 years | 55.51 | [54.95, 56.07] | 70.20 | [69.66, 70.73] |
| 35 years and above | 49.96 | [48.79, 51.13] | 65.84 | [64.65, 67.01] |
| **Mother’s height, *%*** |  |  |  |  |
| <145 cm | 59.76 | [58.49, 61.01] | 72.92 | [71.89, 73.92] |
| >145 cm | 57.57 | [57.09, 58.04] | 71.22 | [70.74, 71.70] |
| **Mother's schooling, *y*** |  |  |  |  |
| No schooling | 55.48 | [54.74, 56.22] | 68.80 | [67.98, 69.60] |
| 1-5 years | 63.43 | [62.38, 64.46] | 73.83 | [72.87, 74.77] |
| 6-8 years | 63.47 | [62.58, 64.36] | 74.94 | [74.11, 75.76] |
| 9-10 years | 62.17 | [61.24, 63.09] | 75.52 | [74.75, 76.28] |
| 11-12 years | 55.16 | [53.96, 56.36] | 72.54 | [71.56, 73.49] |
| 12+ years | 42.13 | [40.79, 43.49] | 62.72 | [61.46, 63.95] |
| **Residence, *%*** |  |  |  |  |
| Urban | 43.15 | [42.06, 44.25] | 59.66 | [58.56, 60.76] |
| Rural | 63.62 | [63.15, 64.09] | 75.63 | [75.16, 76.09] |
| **Health insurance, *%*** |  |  |  |  |
| No | 54.66 | [54.13, 55.19] | 69.29 | [68.72, 69.86] |
| Yes | 68.08 | [67.26, 68.89] | 75.18 | [74.58, 75.77] |
| **Household size** , ***n*** |  |  |  |  |
| <5 members | 58.48 | [57.71, 59.26] | 72.01 | [71.17, 72.84] |
| >5 members | 57.53 | [57.02, 58.03] | 71.16 | [70.67, 71.65] |
| **Religion, *%*** |  |  |  |  |
| Others | 62.72 | [59.91, 65.45] | 65.19 | [62.95, 67.38] |
| Hindu | 59.30 | [58.78, 59.81] | 72.65 | [72.16, 73.13] |
| Muslim | 49.85 | [48.51, 51.19] | 66.25 | [64.88, 67.60] |
| Christian | 58.68 | [55.78, 61.53] | 71.29 | [69.18, 73.31] |
| **Caste category, *%*** |  |  |  |  |
| General | 50.93 | [49.89, 51.97] | 65.86 | [64.78, 66.92] |
| Scheduled caste | 63.15 | [62.15, 64.14] | 74.53 | [73.76, 75.28] |
| Scheduled tribes | 68.00 | [66.88, 69.11] | 78.80 | [77.67, 79.89] |
| Other backward classes | 56.53 | [55.88, 57.17] | 70.96 | [70.35, 71.56] |
| **Wealth quintile, *1-5*** |  |  |  |  |
| Lowest | 59.56 | [58.77, 60.34] | 73.35 | [72.59, 74.09] |
| Second | 64.96 | [64.19, 65.73] | 75.33 | [74.59, 76.06] |
| Middle | 63.79 | [62.95, 64.63] | 75.82 | [74.99, 76.62] |
| Fourth | 55.67 | [54.58, 56.76] | 71.43 | [70.51, 72.34] |
| Highest | 38.63 | [37.49, 39.79] | 57.59 | [56.39, 58.77] |
| **India** | **57.80** | **[57.33, 58.26]** | **71.40** | **[70.95, 71.86]** |
|  | | | | |

**Note.** Q1 refers to the bottom wealth quintile and Q5 refers to the top wealth quintile. All estimates are adjusted for sampling weights.

**Table S3. Prevalence of underweight among Indian children aged 6-59 months stratified by covariates for the period 2015-16 to 2019-21**

|  | | | | |
| --- | --- | --- | --- | --- |
| **Covariates** | **NFHS-4 (2015-16)** | | **NFHS-5 (2019-21)** | |
|  | **Prevalence (%)** | **95% CI** | **Prevalence (%)** | **95% CI** |
| **Child age, *months*** |  |  |  |  |
| 6-23 months | 33.64 | [33.13, 34.15] | 28.52 | [28.02, 29.04] |
| 24-35 months | 38.03 | [37.38, 38.68] | 32.92 | [32.24, 33.61] |
| 36-59 months | 38.78 | [38.28, 39.28] | 33.52 | [33.02, 34.02] |
| **Child’s sex, *%*** |  |  |  |  |
| Male | 36.36 | [35.90, 36.82] | 31.03 | [30.55, 31.51] |
| Female | 37.31 | [36.86, 37.76] | 32.39 | [31.95, 32.84] |
| **Birth order, *%*** |  |  |  |  |
| 1 | 32.35 | [31.84, 32.86] | 27.57 | [27.08, 28.05] |
| 2 | 35.94 | [35.37, 36.52] | 31.23 | [30.69, 31.77] |
| 3+ | 43.96 | [43.40, 44.52] | 38.35 | [37.74, 38.96] |
| **Mother's age, *y*** |  |  |  |  |
| 15-24 years | 36.93 | [36.36, 37.50] | 31.81 | [31.23, 32.40] |
| 25-34 years | 36.24 | [35.81, 36.67] | 31.56 | [31.12, 32.00] |
| 35 years and above | 40.74 | [39.70, 41.79] | 32.7 | [31.65, 33.78] |
| **Mother’s height, *%*** |  |  |  |  |
| <145 cm | 53.15 | [52.03, 54.26] | 46.94 | [45.95, 47.94] |
| >145 cm | 34.72 | [34.35, 35.08] | 29.68 | [29.31, 30.06] |
| **Mother's schooling, *y*** |  |  |  |  |
| No schooling | 48.27 | [47.70, 48.84] | 42.28 | [41.56, 43.00] |
| 1-5 years | 41.21 | [40.29, 42.14] | 36.74 | [35.85, 37.63] |
| 6-8 years | 36.72 | [35.94, 37.50] | 33.4 | [32.62, 34.19] |
| 9-10 years | 30.63 | [29.85, 31.41] | 29.46 | [28.77, 30.16] |
| 11-12 years | 26.71 | [25.78, 27.65] | 25.29 | [24.48, 26.11] |
| 12+ years | 19.9 | [19.01, 20.82] | 19.75 | [18.75, 20.79] |
| **Residence, *%*** |  |  |  |  |
| Urban | 30.2 | [29.42, 31.0] | 26.57 | [25.81, 27.34] |
| Rural | 39.5 | [39.14, 39.87] | 33.59 | [33.20, 33.99] |
| **Health insurance, *%*** |  |  |  |  |
| No | 37.93 | [37.54, 38.32] | 32.72 | [32.28, 33.17] |
| Yes | 33.35 | [32.66, 34.04] | 29.97 | [29.46, 30.49] |
| **Household size**, ***n*** |  |  |  |  |
| <5 members | 34.62 | [33.98, 35.27] | 30.53 | [29.87, 31.19] |
| >5 members | 37.74 | [37.34, 38.13] | 32.21 | [31.82, 32.61] |
| **Religion, *%*** |  |  |  |  |
| Others | 32.35 | [30.50, 34.25] | 26.13 | [24.37, 27.96] |
| Hindu | 37.41 | [37.03, 37.79] | 31.89 | [31.50, 32.28] |
| Muslim | 36.11 | [35.20, 37.02] | 32.65 | [31.73, 33.58] |
| Christian | 28.41 | [26.41, 30.50] | 25.13 | [23.43, 26.92] |
| **Caste category, *%*** |  |  |  |  |
| General | 29.91 | [29.12, 30.72] | 26.19 | [25.45, 26.95] |
| Scheduled caste | 40.25 | [39.52, 40.97] | 34.98 | [34.28, 35.69] |
| Scheduled tribes | 47.17 | [46.26, 48.08] | 39.49 | [38.56, 40.43] |
| Other backward classes | 36.43 | [35.95, 36.92] | 30.9 | [30.39, 31.41] |
| **Wealth quintile, *1-5*** |  |  |  |  |
| Lowest | 50.4 | [49.79, 51.00] | 43.51 | [42.84, 44.17] |
| Second | 41.74 | [41.08, 42.39] | 35.48 | [34.82, 36.14] |
| Middle | 34.64 | [33.90, 35.39] | 30.26 | [29.55, 30.98] |
| Fourth | 27.89 | [27.07, 28.72] | 24.68 | [23.94, 25.42] |
| Highest | 21.63 | [20.78, 22.50] | 19.1 | [18.22, 20.02] |
| **India** | **36.86** | **[36.51, 37.20]** | **31.74** | **[31.38, 32.09]** |
|  | | | | |

**Note.** Q1 refers to the bottom wealth quintile and Q5 refers to the top wealth quintile. All estimates are adjusted for sampling weights.

**Table S4: Testing for parallel trends using placebo treatment run on 2005-06 and 2015-16 (pre-intervention period)**

| **Outcome=Underweight*, binary*** | **Coefficient** | **95% CI** | **P-value** |
| --- | --- | --- | --- |
| **Model1: Any service under ICDS** | | | |
| Placebo treatment*, binary* | 0.07 | [0.03, 0.11] | 0.00 |
| Linear trend, *%* | 0.00 | [0.00, 0.00] | 0.97 |
| Interaction, *%* | 0.00 | [-0.01, 0.00] | 0.15 |
| Intercept | 0.33 | [0.30, 0.36] | 0.00 |
| **Model2: Any service other than food supplementation** | | | |
| Placebo treatment*, binary* | 0.09 | [0.06, 0.13] | 0.00 |
| Linear trend, *%* | 0.00 | [0.00, 0.00] | 0.93 |
| Interaction, *%* | 0.00 | [-0.01, 0.00] | 0.02 |
| Intercept | 0.33 | [0.30, 0.36] | 0.00 |
| **Model3: Food supplementation** | | | |
| Placebo treatment*, binary* | 0.07 | [0.02, 0.11] | 0.00 |
| Linear trend, *%* | 0.00 | [0.00, 0.00] | 0.76 |
| Interaction, *%* | 0.00 | [-0.01, 0.00] | 0.05 |
| Intercept | 0.34 | [0.30, 0.37] | 0.00 |

**Note.** The parallel trends compare children aged 6-59 months in 2005-2006 (pre-intervention period) with children in 2015-16. Placebo treatment is for the following services availed for children aged 6-59 months- 1) Any service under ICDS in Model1, 2) non-food services from ICDS in Model2, 3) food-based services from ICDS in Model3; Linear trend= linear variable for time, takes value 1 for 2005-06 and 10 for 2015-16. Interaction=interaction between the placebo treatment and trend variable. Estimates were calculated using ordinary least squares model where outcome is underweight and standard errors are clustered at state.

**Table S5:** Interrupted time series model testing for accelerating trends among children receiving any child specific service under ICDS

| **Underweight, *binary*** | **Coefficient** | **P-value** | **95% CI** |
| --- | --- | --- | --- |
| Linear trend, *%* | -0.003 | 0.000 | [-0.004, -0.002] |
| Post treatment period*linear trend, *%* | -0.003 | 0.000 | [-0.004, -0.003] |
| Intercept | 0.404 | 0.000 | [0.393, 0.415] |

**Note.** The interrupted time series model checks for accelerating trend in underweight reduction among children aged 6-59 months receiving ICDS in 2015-16. The interaction term is significant supporting acceleration of trend i.e. reduction in underweight among children receiving ICDS. Linear trend= linear variable for time, takes value 1 for 2005-06, 10 for 2015-16, 15 for 2019-21. Post treatment period= dummy variable for the survey year 2019-21. Estimates were calculated using a ordinary least squares model where outcome is underweight. Standard errors are clustered at state. This regression was run on a sub-sample of children who received any ICDS treatment.

**Table S6: Individual-level analysis: Regression based difference-in-differences estimates for association between receiving services from ICDS and stunting prevalence among children aged 6-59 months, 2015-2021**

|  | Stunting prevalence, percentage points | | | |
| --- | --- | --- | --- | --- |
|  | All India sample | | Unsplit districts sub-sample | |
|  | Beta | 95% CI | Beta | 95% CI |
| **Model 1: Any ICDS service** | | | | |
| Post treatment period, *%* | -2.32* | [-3.12,-1.52] | -2.47* | [-3.32,-1.61] |
| Treatment group, *%* | 1.70* | [1.17,2.23] | 1.67* | [1.12,2.21] |
| Interaction (DID), *%* | -0.10 | [-0.89,0.70] | -0.34 | [-1.19,0.50] |
| **Model 2: Non-food ICDS services** | | | | |
| Post treatment period, *%* | -2.06* | [-2.88,-1.25] | -2.27* | [-3.12,-1.43] |
| Treatment group, *%* | 1.75* | [1.21,2.29] | 1.73* | [1.18,2.28] |
| Interaction (DID), *%* | -0.51 | [-1.33,0.32] | -0.68 | [-1.52,0.16] |
| **Model3: Food based ICDS services** | | | | |
| Post treatment period, *%* | -2.62* | [-3.38,-1.86] | -2.84* | [-3.65,-2.03] |
| Treatment group, *%* | 1.39* | [0.88,1.91] | 1.36* | [0.83,1.88] |
| Interaction (DID), *%* | 0.36 | [-0.41,1.13] | 0.21 | [-0.61,1.02] |
| **Model specifications** |  |  |  |  |
| All covariates | Yes |  | Yes |  |
| District fixed effects | Yes |  | Yes |  |
| Clustered standard errors at district level |  | Yes |  | Yes |
| N (number of children) | 388252 |  | 336959 |  |

**Note.** ^*^ *p* < 0.05, ^**^ *p* < 0.01, ^***^ *p* < 0.001; Post treatment period=dummy variable for 2019-21; Treatment group refers to any ICDS in model1, non-food services from ICDS in model2, food-based services from ICDS in model3; DID=difference-in-differences estimate; Estimates are calculated using ordinary least squares model where outcome is underweight (see equation 2). Binary outcome was multiplied by 100 and can be interpreted as a percentage point difference. Covariates include child age, sex, birth order, maternal age, maternal height, maternal education, household residence, health insurance, household size, religion, caste and wealth. Coefficients not shown for brevity.

**Table S7: Individual-level analysis: Regression based difference-in-differences estimates for association between receiving services from ICDS and wasting prevalence among children aged 6-59 months, 2015-2021**

|  | Wasting prevalence, percentage points | | | |
| --- | --- | --- | --- | --- |
|  | All India sample | | Unsplit districts sub-sample | |
|  | Beta | 95% CI | Beta | 95% CI |
| **Model 1: Any ICDS service** | | | | |
| Post treatment period, *%* | -0.98* | [-1.84,-0.12] | -0.84 | [-1.78,0.10] |
| Treatment group, *%* | 0.96* | [0.47,1.44] | 1.08* | [0.58,1.59] |
| Interaction (DID), *%* | -0.91* | [-1.62,-0.20] | -1.04* | [-1.82,-0.27] |
| **Model 2: Non-food ICDS services** | | | | |
| Post treatment period, *%* | -0.83 | [-1.69,0.02] | -0.70 | [-1.62,0.23] |
| Treatment group, *%* | 1.11* | [0.61,1.60] | 1.27* | [0.75,1.78] |
| Interaction (DID), *%* | -1.21* | [-1.94,-0.48] | -1.35* | [-2.15,-0.56] |
| **Model3: Food based ICDS services** | | | | |
| Post treatment period, *%* | -1.01* | [-1.86,-0.17] | -0.89 | [-1.80,0.03] |
| Treatment group, *%* | 1.15* | [0.66,1.64] | 1.30* | [0.79,1.81] |
| Interaction (DID), *%* | -1.01* | [-1.73,-0.29] | -1.14* | [-1.92,-0.37] |
| **Model specifications** |  |  |  |  |
| All covariates | Yes |  | Yes |  |
| District fixed effects | Yes |  | Yes |  |
| Clustered standard errors at district level | Yes |  | Yes |  |
| N (number of children) | 388956 |  | 337712 |  |

**Note.** ^*^ *p* < 0.05, ^**^ *p* < 0.01, ^***^ *p* < 0.001; Post treatment period=dummy variable for 2019-21; Treatment group refers to any ICDS in model1, non-food services from ICDS in model2, food-based services from ICDS in model3; DID=difference-in-differences estimate; Estimates are calculated using ordinary least squares model where outcome is underweight (see equation 2). Binary outcome was multiplied by 100 and can be interpreted as a percentage point difference. Covariates include child age, sex, birth order, maternal age, maternal height, maternal education, household residence, health insurance, household size, religion, caste and wealth. Coefficients not shown for brevity.

**Table S8: Individual-level analysis: Sensitivity analysis for difference-in-differences models using sub-samples of states surveyed before and during COVID-19**

|  | Underweight prevalence, percentage points | | | |
| --- | --- | --- | --- | --- |
|  | Surveyed before COVID-19 | | Surveyed during COVID-19 | |
|  | Beta | 95% CI | Beta | 95% CI |
| **Model 1: Any ICDS service** | | | | |
| Post treatment period, *%* | -0.50 | [-1.59,0.59] | -7.03* | [-8.09,-5.97] |
| Treatment group, *%* | 1.69^*^ | [0.89,2.50] | 1.87* | [1.18,2.56] |
| Interaction (DID), *%* | -0.49 | [-1.67,0.69] | -1.02* | [-2.00,-0.04] |
| **Model 2: Non-food ICDS services** | | | | |
| Post treatment period, *%* | 0.03 | [-1.14,1.20] | -7.13* | [-8.17,-6.08] |
| Treatment group, *%* | 1.97* | [1.14,2.80] | 1.85* | [1.16,2.54] |
| Interaction (DID), *%* | -1.40* | [-2.66,-0.15] | -0.93 | [-1.88,0.03] |
| **Model3: Food based ICDS services** | | | | |
| Post treatment period, *%* | -1.15* | [-2.18,-0.13] | -7.10* | [-8.14,-6.06] |
| Treatment group, *%* | 1.08* | [0.29,1.87] | 1.82* | [1.12,2.52] |
| Interaction (DID), *%* | 0.54 | [-0.57,1.66] | -1.03* | [-2.02,-0.05] |
| **Model specifications** |  |  |  |  |
| All covariates | Yes |  | Yes |  |
| District fixed effects | Yes |  | Yes |  |
| Clustered standard errors at district level |  | Yes |  | Yes |
| N (number of children) | 177172 |  | 217901 |  |

**Note.** ^*^ *p* < 0.05, ^**^ *p* < 0.01, ^***^ *p* < 0.001; Post treatment period=dummy variable for 2019-21; Treatment group refers to any ICDS in model1, non-food services from ICDS in model2, food-based services from ICDS in model3; DID=difference-in-differences estimate; Estimates are calculated using ordinary least squares model where outcome is underweight (see equation 2). Binary outcome was multiplied by 100 and can be interpreted as a percentage point difference. Covariates include child age, sex, birth order, maternal age, maternal height, maternal education, household residence, health insurance, household size, religion, caste and wealth. Coefficients not shown for brevity. The states surveyed during or after COVID-19 include Punjab, Chandigarh, Uttarakhand, Haryana, NCT of Delhi, Rajasthan, Uttar Pradesh, Arunachal Pradesh, Jharkhand, Odisha, Chhattisgarh, Madhya Pradesh, Tamil Nadu, Puducherry.

**Table S9: Change in coverage of food supplementation service under ICDS between 2015 and 2021.**

|  | **2015-16** | | **2019-21** | |
| --- | --- | --- | --- | --- |
| **Frequency of receiving food supplementation** | ***%,* Coverage** | **95% CI** | ***%,* Coverage** | **95% CI** |
| Not at all | 9.96 | [9.67, 10.27] | 7.07 | [6.83, 7.33] |
| Almost daily | 36.66 | [36.10, 37.21] | 35.32 | [34.82, 35.83] |
| At least once a week | 21.58 | [21.16, 22.02] | 19.66 | [19.30, 20.03] |
| At least once a month | 26.96 | [26.50, 27.43] | 32 | [31.53, 32.48] |
| Less often | 4.58 | [4.40, 4.76] | 5.67 | [5.47, 5.87] |
| Don't know | 0.26 | [0.23, 0.30] | 0.27 | [0.24, 0.32] |
| N (number of children) | 119614 |  | 136103 |  |

**Note.** ICDS=Integrated Child Development Services. All estimates are adjusted for sampling weights.

**Table S10: Individual-level analysis: Robustness checks for the difference-in-differences model using food supplementation frequencies as treatments.**

|  | Underweight prevalence, percentage points | | | | | |
| --- | --- | --- | --- | --- | --- | --- |
|  | All sample | | Surveyed before COVID-19 | | Surveyed during COVID-19 | |
|  | Beta | 95% CI | Beta | 95% CI | Beta | 95% CI |
| Post treatment period, *%* | -4.23^*^ | [-4.99,-3.47] | -1.01 | [-2.05,0.03] | -6.94^*^ | [-7.94,-5.94] |
| Treatment group (frequent food supplementation), *%* | 1.97^*^ | [1.35,2.60] | 1.83^*^ | [0.87,2.80] | 2.12^*^ | [1.31,2.92] |
| Treatment group (less frequent food supplementation), *%* | 1.04^*^ | [0.34,1.74] | 0.65 | [-0.30,1.60] | 1.70^*^ | [0.76,2.64] |
| DID (frequent food supplementation frequently), *%* | -0.97^*^ | [-1.84,-0.09] | -0.38 | [-1.64,0.88] | -1.36^*^ | [-2.53,-0.20] |
| DID (less frequent food supplementation), *%* | 0.07 | [-0.91,1.05] | 1.30 | [-0.15,2.75] | -0.84 | [-2.07,0.38] |
| **Model specifications** |  |  |  |  |  |  |
| All covariates | Yes |  | Yes |  | Yes |  |
| District fixed effects | Yes |  | Yes |  | Yes |  |
| Clustered standard errors at district level | Yes |  | Yes |  | Yes |  |
| N (number of children) | 395073 |  | 177172 |  | 217901 |  |

**Note.** ^*^ *p* < 0.05, ^**^ *p* < 0.01, ^***^ *p* < 0.001; Post treatment period=dummy variable for 2019-21; Treatment group (frequent food supplementation) refers to children who received food supplementation almost daily or at least once a week; Treatment group (less-frequent food supplementation) refers to children who received food supplementation at least once a month or less often; DID=difference-in-differences estimate; Estimates are calculated using ordinary least squares model where outcome is underweight (see equation 2). Binary outcome was multiplied by 100 and can be interpreted as a percentage point difference. Covariates include child age, sex, birth order, maternal age, maternal height, maternal education, household residence, health insurance, household size, religion, caste and wealth. Coefficients not shown for brevity. The states surveyed during or after COVID-19 include Punjab, Chandigarh, Uttarakhand, Haryana, NCT of Delhi, Rajasthan, Uttar Pradesh, Arunachal Pradesh, Jharkhand, Odisha, Chhattisgarh, Madhya Pradesh, Tamil Nadu, Puducherry.

**Table S11: Individual-level analysis: Additional analysis for difference-in-differences models using sub-samples of urban and rural areas.**

|  | Underweight prevalence, percentage points | | | |
| --- | --- | --- | --- | --- |
|  | Rural | | Urban | |
|  | Beta | 95% CI | Beta | 95% CI |
| **Treatment: Any ICDS service** | | | | |
| Post treatment period, *%* | -4.05^*^ | [-4.97,-3.14] | -3.73^*^ | [-4.99,-2.47] |
| Treatment group, *%* | 1.71^*^ | [1.10,2.32] | 1.63^*^ | [0.67,2.58] |
| Interaction (DID), *%* | -1.02^*^ | [-1.89,-0.15] | 0.05 | [-1.40,1.50] |
| **Model specifications** |  |  |  |  |
| All covariates | Yes |  | Yes |  |
| District fixed effects | Yes |  | Yes |  |
| Clustered standard errors at district level |  | Yes |  | Yes |
| N (number of children) | 306981 |  | 88092 |  |

**Note.** ^*^ *p* < 0.05, ^**^ *p* < 0.01, ^***^ *p* < 0.001; Post treatment period=dummy variable for 2019-21; Treatment group refers to any; DID=difference-in-differences estimate; Estimates are calculated using ordinary least squares model where outcome is underweight (see equation 2). Binary outcome was multiplied by 100 and can be interpreted as a percentage point difference. Covariates include child age, sex, birth order, maternal age, maternal height, maternal education, household residence, health insurance, household size, religion, caste and wealth. Coefficients not shown for brevity.
